# Classification of the Confocal Microscopy Images of Colorectal Tumor and Inflammatory Colitis Mucosa Tissue Using Deep Learning

**DOI:** 10.3390/diagnostics12020288

**Published:** 2022-01-24

**Authors:** Jaehoon Jeong, Seung Taek Hong, Ihsan Ullah, Eun Sun Kim, Sang Hyun Park

**Affiliations:** 1 Department of Robotics Engineering, Daegu Gyeongbuk Institute of Science and Technology (DGIST), 333 Techno Jungang-Daero, Dalseong-gun, Daegu 42988, Korea; j.hoon@dgist.ac.kr (J.J.); ihsankhan@dgist.ac.kr (I.U.); 2 Institute of Gastrointestinal Medical Instrument Research, Korea University College of Medicine, 73 Inchon-ro, Seongbuk-gu, Seoul 02841, Korea; ikoiko7@korea.ac.kr; 3 Department of Internal Medicine, Korea University College of Medicine, 73 Inchon-ro, Seongbuk-gu, Seoul 02841, Korea

**Keywords:** colorectal neoplasm, colorectal inflammation, confocal microscopy, deep learning, machine learning

## Abstract

Confocal microscopy image analysis is a useful method for neoplasm diagnosis. Many ambiguous cases are difficult to distinguish with the naked eye, thus leading to high inter-observer variability and significant time investments for learning this method. We aimed to develop a deep learning-based neoplasm classification model that classifies confocal microscopy images of 10× magnified colon tissues into three classes: neoplasm, inflammation, and normal tissue. ResNet50 with data augmentation and transfer learning approaches was used to efficiently train the model with limited training data. A class activation map was generated by using global average pooling to confirm which areas had a major effect on the classification. The proposed method achieved an accuracy of 81%, which was 14.05% more accurate than three machine learning-based methods and 22.6% better than the predictions made by four endoscopists. ResNet50 with data augmentation and transfer learning can be utilized to effectively identify neoplasm, inflammation, and normal tissue in confocal microscopy images. The proposed method outperformed three machine learning-based methods and identified the area that had a major influence on the results. Inter-observer variability and the time required for learning can be reduced if the proposed model is used with confocal microscopy image analysis for diagnosis.

## 1. Introduction

Colorectal cancer (CRC) is the second deadliest cancer worldwide [1]. The precise and timely diagnosis of CRC is critical for improving treatment efficacy. However, conventional CRC diagnosis requires thorough visual examinations by highly experienced endoscopists. Conventional approaches [2,3,4,5] toward CRC management include the sampling of suspicious lesions via regular colonoscopy and deciding future countermeasures via histological analysis [6,7]. Consequently, conventional CRC diagnosis requires a long time for proper examination (ranging from several days to weeks); if an abnormality is identified, it is cumbersome to repeat the secondary endoscopic examination [7,8]. Moreover, endoscopists are required to have high concentration during examinations to avoid possible errors. Therefore, it is imperative to develop a reliable system for CRC analysis that can improve clinical efficiency and minimize potential errors during diagnosis.

In recent years, there has been increased interest worldwide in exploring methods for the prevention, diagnosis, and improved visualization of CRC [2,3,4,5]. Most techniques [9,10,11,12] integrate confocal laser endomicroscopy (CLE) and narrow banding imaging into the tip of a flexible endoscope to provide high-resolution endomicroscopy for real-time virtual biopsies. For example, the Cellvizio probe-based CLE has been commercialized. However, CRC diagnosis with such a system remains difficult because of the long processing time, inconsistent efficiency, physician disagreement, long learning curves, and low consistency across observers [13,14,15]. To address this issue, computer-assisted assessment that uses artificial intelligence technology has recently been explored extensively for lesion identification [5,16,17,18].

Specifically, machine learning and deep learning-based methods have been proposed [2,19,20,21]. Tamaki et al. [20] extracted image features by using the bag-of-visual-words method and then classified colorectal tumor images into three tumor types (A, B, and C3) by using the support vector machine (SVM) classifier. Zhou et al. [19] developed a dense convolutional network by using colonoscopic images to classify CRC from normal tissues. To supplement the small training data, Kolligs [2] and Ito et al. [2,21] adopted a deep transfer learning method that pre-trained the model with ImageNet and updated the last layer of the model with CRC data. However, even though these studies achieved high accuracy scores on classification tasks, the scores were not compared with the predictions by endoscopists, thus making it difficult to determine the difficulty of the task they achieved. Furthermore, most methods primarily focused on CRC classification, with relatively little attention paid to inflammatory bowel disease (IBD) [22]. The number of patients with IBD, which has recently established itself as a global disease, is rapidly increasing [23,24,25,26]. However, it is difficult to accurately distinguish CRC from colonic inflammation because the patterns appearing in tissue confocal microscopy images look similar [27,28,29,30]. To the best of our knowledge, no study has classified CRC and IBD by using a machine learning-based method. Therefore, this study presents a deep learning method for classifying confocal microscopy images into colorectal neoplasms, colon inflammation, and normal tissues. We collected 411 confocal microscopy images from normal, CRC, and IBD tissues that were subjected to histological analysis and then trained and tested a deep learning model to classify them with a 4-fold cross-validation setting. We then compared the performance of the proposed model with those of three machine learning-based methods by using radiomic features and the predictions of four endoscopists.

## 2. Materials and Methods

### 2.1. Dataset

Fresh colon tissues were collected from 29 individuals who were over 18 years old, provided informed consent, and underwent elective colonoscopies at Korea University Medical Center, Anam Hospital, by following a protocol approved by the institutional review board (2019AN0051). Bright field images of the tissues were obtained using a confocal microscope (Leica TCS SP2, Leica, Solms, Germany) with a mode-locked Ti:sapphire laser source (Chameleon, Coherent Inc., Santa Clara, CA, USA) set at a wavelength of 750 nm and with a 10× dry objective with a numerical aperture of 0.30. A total of 132 ex vivo colon tissues were obtained from 29 patients, including 68 normal colon tissues, 18 inflamed colon tissues, and 46 neoplasm colon tissues. Tissue types were determined using histological analysis, which is the gold standard of diagnosis of colon tumor/inflammation/normal. From the tissues, we imaged 411 confocal microscopy images, including 178 normal images, 173 tumor images, and 60 inflammatory images.

### 2.2. Assessment by Endoscopists

Four endoscopists performed an anonymous evaluation of each confocal microscopy image of colon biopsy sample to determine whether it was a neoplasm, inflammation, or normal tissue. The four endoscopists were all experts and had performed more than 200 colonoscopies. The colon images were evaluated anonymously, and there was no communication among the endoscopists regarding the classification.

### 2.3. Classification Using Machine Learning Methods

We extracted image features from the confocal microscopy images and utilized them as inputs for the three machine learning methods. Specifically, the PyRadiomics toolbox [31] was used to extract radiomic features [32] from the collected colon images. We extracted first-order statistical features that describe the distribution of individual pixel values, as well as second-order statistical features (also called textural features), and higher-order statistical features by using statistical methods after applying filters or mathematical transforms to the images [33]. Among the extracted features, we selected some important features to exclude junk information on classification. To determine which feature is fundamental to classification, we computed importance scores by using a machine learning library called scikit-learn. By referring to the importance score, we empirically selected the top 94 important features and used them for classification. Features related to gray-level intensity and textures, such as gray-level non-uniformity, short-run emphasis, and zone percentage feature, were highly selected rather than first-order features. By using the selected radiomic features, we trained three classification models: random forest (RF) [34], SVM [35], and extreme gradient boost (XGB) [36].

### 2.4. Classification Using Deep Convolutional Neural Networks

Unlike the machine learning methods using hand crafted features, we can learn the relationship between images and labels in end-to-end manner via deep convolutional neural networks. We trained a deep learning model by using the residual network [37], which is known to achieve high performance in various image classification tasks. We used data augmentation and transfer learning to handle the lack of annotated colon data. Specifically, we sampled 20 images during each mini-batch training and transformed them with random horizontal and vertical flips and random rotations between −180° and 180°. Data augmentation was also performed on machine learning-based methods for fair comparisons. For transfer learning, we used the ImageNet pre-trained model and then froze the model except for the last layer. We additionally trained the model and updated the last layer with confocal colorectal images.

**Figure 1:**
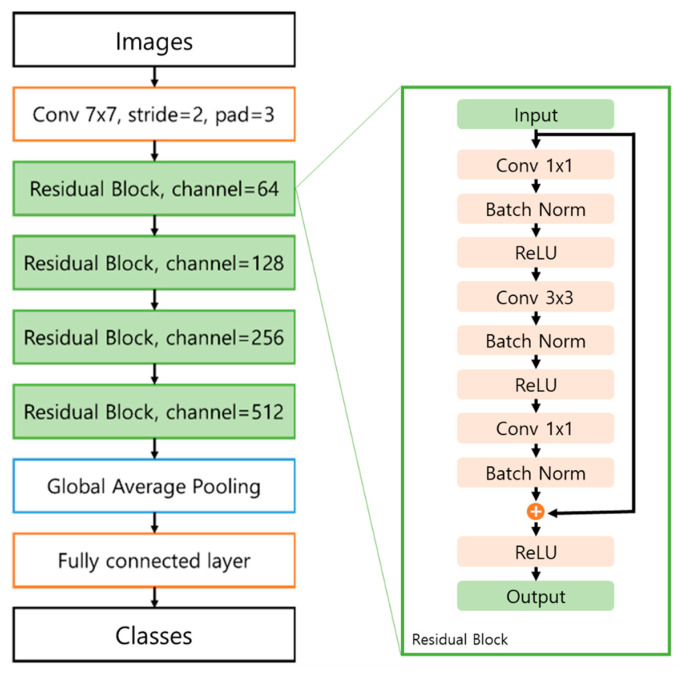
The proposed residual network architecture. Conv, pad, Batch Norm, and ReLU indicates convolution, padding, batch normalization [40], and rectified linear unit [38], respectively.

The residual network consists of four residual blocks including skip connections, a global average pooling (GAP) layer, and a fully connected layer (Figure 1). Convolutional layers and max pooling layers were used to extract informative features from images and prominent signals from the feature maps. We used 3 × 3 filters for each convolution layer and max pooling layer and used batch normalization after each convolution layer. To extract the prominent signals, the pooling layer downsamples the image to a small size. When the image size was reduced by the pooling and convolution layers, the number of channels gradually doubled from 64 to 512. A rectified linear unit (ReLU) [38], alleviating the gradient vanishing problem, was used as the activation function for all the convolution layers. The ResNet50 model applied the GAP [39] layer at the end of the model, which can conserve location information. The conserved location information can then be used to visualize the influential area in the testing stage.

To train the proposed model, the mini-batch size was fixed to 20. The error between the label and prediction was calculated using cross-entropy with the softmax function. The model was optimized by the ADAM optimizer [41]. The learning rate was fixed to 0.001 with a total of 100 epochs. Experiments were performed using a PC equipped with an Intel i7-8700K 3.7 GHz CPU, an NVIDIA GTX 1080 Ti GPU, and 64 GB of RAM, and the algorithms were implemented in PyTorch [42].

During the testing stage, the proposed model computes the prediction score from a given colorectal image. We employ class activation maps (CAMs) [43], which visually activate the parts that have a significant influence on the classification. To construct the CAM, we retrieved the GAP layer of the trained network that conserved the location information. By using the location information, we obtained a map of the most salient features used in classifying the image pixels as neoplasms.

### 2.5. Evaluation Settings

For evaluation, we performed 4-fold cross-validation by dividing 411 images into 4 sets (namely, 102, 103, 103, and 103), and each set had an equal distribution of the 3 classes. We used 3 of the 4 sets as training data and 1 set as test data, and the aforementioned process was repeated for every fold to obtain the prediction scores of all 411 images. A similar procedure was repeated for the machine learning-based methods with the same data splits.

We evaluated the performance in terms of accuracy, precision, recall, F1-score, false positive rate (FPR), and false negative rate (FNR). Moreover, to demonstrate the effectiveness of the proposed method, we compared its performance to the assessments of 4 endoscopists for all 411 images. We computed confusion matrices to check for inter-observer variability among the assessments of the four endoscopists and highlighted the efficacy of the proposed method over the assessments. To confirm the robustness of the proposed method, we investigated the prediction trends by evaluating the FPR and FNR.

## 3. Results

### 3.1. Performance of Predictions by the Endoscopists

Table 1 and Table 2 show the classification scores of the four endoscopists. The accuracy scores of the four endoscopists were 0.51, 0.62, 0.59, and 0.64. The average accuracy of the four endoscopists on normal images was 72% even though 14% of images were misinterpreted as tumors and inflammation. They predicted 55% of tumor images well, but 38% were incorrectly predicted as inflammation. For inflammation images, 34% were well predicted, but 53% of images were incorrectly classified as tumors.

### 3.2. Comparison of Learning-Based Methods

Table 3 and Table 4 present the four fold cross-validation performance of the three machine learning-based approaches and the proposed deep learning-based method. Among the machine learning-based methods, XGB achieved the highest score in all performance metrics. The performance difference between RF and SVM was insignificant, but SVM performed the worst in terms of accuracy, precision, and F1-scores. The proposed method achieved state of the art results and outperformed all the machine learning-based methods. The proposed method has a high accuracy of 81.73% in categorizing confocal microscopy images of colon biopsy samples into three categories. After comparing the results of the proposed method with those of the machine learning-based methods, we found a marked improvement of +9.21% in accuracy on average. Moreover, the proposed method obtained the lowest FPR and FNR (lower FPR and lower FNR are better).

**Figure 2:**
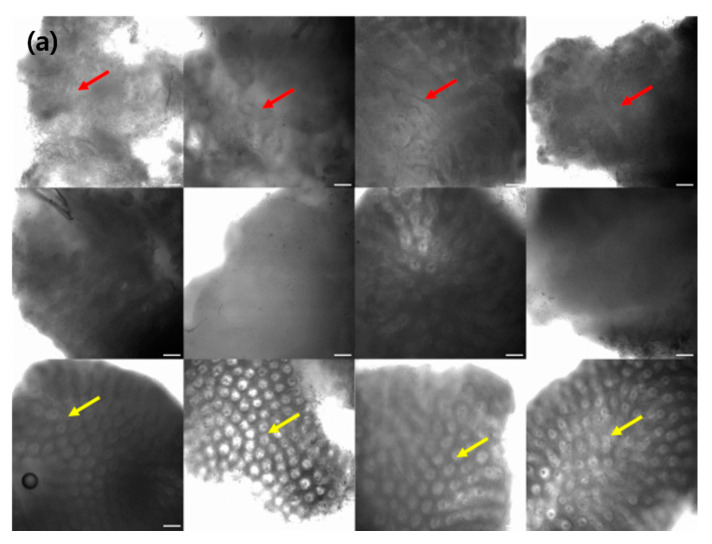

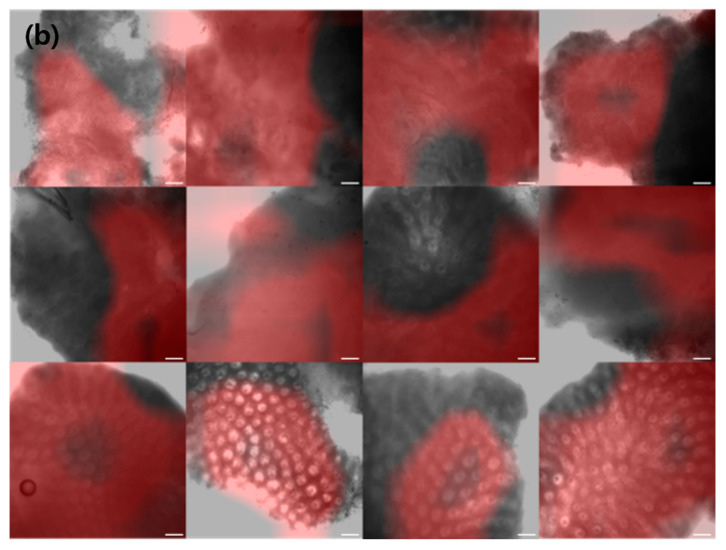
Test data samples (**a**) and the results of class activation map (**b**). Neoplasm examples are shown in the top row, inflammation in the middle row, and normal in the bottom row, respectively. Red arrows on neoplasm images indicate the area where the glands are hard to see, and yellow arrows on normal images indicate where the glands are uniformly represented. The arrangement of gland is an important feature on neoplasm classification task, and it can be seen that CAM focuses well on this area. The white bars on the lower right of the image indicates the scale 50 μm.

To facilitate endoscopists in the clinical diagnosis of CRC, we employed a CAM, which activates the regions that are crucial for CRC categorization in a confocal microscopy image. Figure 2 depicts various examples of normal, tumor, and inflammation-activated regions using CAMs. A healthy normal colon mucosa is characterized by dark goblet cells, regular and narrow vessels surrounding crypts, and a round crypt structure [44], whereas inflammation of the colon mucosa is characterized by variations in the shape, size, and distribution of crypts, increased distance between crypts, focal crypt distribution, mild-to-moderate increase in capillaries, and dilated and distorted crypts [45,46,47]. Neoplasm colon mucosa is characterized by a ridge-lined irregular epithelial layer with the loss of crypts and goblet cells, irregular cell architecture with little or no mucin, dilated and distorted vessels with increased leakage, irregular architecture with little or no orientation to adjunct tissue, disorganized villiform or lack of structure, dark and irregularly thickened epithelium, and dilated vessels [14,48]. Our CAM results show the well-activated characteristic areas of each neoplasm, inflammation, and normal tissue (Figure 2); for example, the activated regular and round crypt structures of neoplasms, dispersed crypt structure of inflammation, and irregular cell architecture of neoplasms.

## 4. Discussion

This study demonstrated the efficacy of the proposed model by comparing it with the performance of three machine learning methods and the predictions of four endoscopists. The proposed method provides a significant performance improvement compared with the XGB, SVM, and RF methods. Machine learning-based methods frequently misclassify normal and tumor classes, which may lead to incorrect treatment for the patients. On the contrary, the proposed deep learning-based method obtained 12% and 17% better accuracy rates for classifying normal and tumor classes in images, respectively. The proposed method also produced better results than the evaluations of the endoscopists in terms of accuracy, precision, recall, and F1 score by +22.60%, +25.14%, +22.26%, and +24.15%, respectively. There was a high degree of inter-observer variability among the decisions of the endoscopists, particularly in differentiating between inflammation and neoplasm classes. For example, endoscopists A and C were inclined to predict neoplasm as inflammation, whereas endoscopists B and D were inclined to predict inflammation as a neoplasm class. Therefore, the classification evaluations of the endoscopists indicate a significant bias and low accuracy in neoplasm–inflammation classification. On the other hand, the proposed method obtained consistent performance in the classification of neoplasm and inflammation. After rigorous evaluation of the performance of the proposed method, we confirmed that the proposed method will perform with high reliability when employed in the clinical workflow for diagnosing neoplasms in colonoscopies.

In the literature, prior studies [49,50] have shown 93.1% and 89.1% accuracy rates for colon classification. However, these techniques only consider binary classification tasks. Our method achieved an accuracy of 81.7% for the three class classification tasks, including the difficult task of distinguishing neoplasm and inflammation classes. In the binary classification problem that classifies CRC and normal tissue, our method can obtain an accuracy of 95.1%.

In medical settings, diagnosis is dependent on the subjective opinions of analysts and often shows a significant discrepancy between experienced and novice endoscopists. We confirmed that the proposed deep learning-based method may assist novice endoscopists in confusing situations, particularly in tumor-inflammation classification. Furthermore, the proposed method can accelerate the analysis. In particular, our proposed method predicts the confocal image class with higher accuracy in less than 1 s, whereas endoscopists often require 5–10 s to diagnose a single confocal image. Moreover, CAM visualization can help novice endoscopists with confocal image interpretation because they are less reliable and take longer to interpret confocal images.

Despite the significant performance of the proposed method across various settings, we highlight some shortcomings and constraints that require careful consideration. First, although data augmentation and transfer learning methods were used, the annotated colon dataset was insufficient in our experiments. Additionally, the current work is limited to the single domain study. In the future, we would like to expand our model to address more general tissue images taken from various hospitals. Since each hospital has different lens specs or laser specs for commercially available confocal microscopes, there may be differences in the images. However, the specifications of the commercial confocal microscope used in this study are not particularly high-level special equipment, and the model will be generalized if more images from various hospitals can be used in the training stage. We will verify the performance of the proposed method on a large amount of data obtained from more sites. Second, the images used in our experiments were acquired using a microscope instead of a probe-based CLE. In the future, we plan to create a system that can assist decision making while viewing the CLE in real time.

## 5. Conclusions

We proposed a colorectal neoplasm classification system that uses a deep learning model with data augmentation and transfer learning to effectively identify neoplasm, inflammation, and normal tissue in confocal microscopy images. The proposed method outperforms the classification accuracy of experienced endoscopists, as well as the accuracy of the three machine learning-based methods. We expect that the proposed deep learning-based method is feasible and capable of assisting endoscopists in decision making with high precision for colorectal neoplasm classification.

## Figures and Tables

**Table 1:** Confusion matrices of four endoscopists. T, I, and N denote tumor, inflammation, and normal, respectively.

**A**	**Label_T**	**Label_I**	**Label_N**	**B**	**Label_T**	**Label_I**	**Label_N**
Pred_T	41	16	18	Pred_T	128	43	15
Pred_I	119	38	28	Pred_I	39	8	43
Pred_N	13	6	132	Pred_N	6	9	120
**C**	**Label_T**	**Label_I**	**Label_N**	**D**	**Label_T**	**Label_I**	**Label_N**
Pred_T	83	26	22	Pred_T	131	43	37
Pred_I	76	29	25	Pred_I	29	7	16
Pred_N	14	5	131	Pred_N	13	10	125

**Table 2:** Classification results of the four endoscopists.

Endoscopist	Accuracy	FPR	FNR	Precision	Recall	F1-Score
A	0.5133	0.2144	0.4627	0.5420	0.5373	0.4810
B	0.6228	0.1805	0.4842	0.5553	0.5257	0.5288
C	0.5912	0.1903	0.4336	0.5766	0.5663	0.5500
D	0.6399	0.1876	0.4746	0.5333	0.5253	0.5247
Endo-AVG	0.5913	0.1932	0.4637	0.5614	0.5362	0.5369

**Table 3:** Confusion matrices of machine learning.

**XGB**	**Label_T**	**Label_I**	**Label_N**	**SVM**	**Label_T**	**Label_I**	**Label_N**
Pred_T	130	11	38	Pred_T	125	18	43
Pred_I	7	43	5	Pred_I	7	37	10
Pred_N	36	6	135	Pred_N	41	5	125
**RF**	**Label_T**	**Label_I**	**Label_N**	**Proposed**	**Label_T**	**Label_I**	**Label_N**
Pred_T	126	15	41	Pred_T	148	22	15
Pred_I	6	33	6	Pred_I	6	31	2
Pred_N	41	12	131	Pred_N	19	7	161

**Table 4:** Machine learning and deep learning performances.

Methods	Accuracy	FPR	FNR	Precision	Recall	F1-Score
XGB	0.7495	0.1401	0.2578	0.7591	0.7423	0.7488
SVM	0.6981	0.1673	0.3195	0.7191	0.6806	0.6786
RF	0.7058	0.1656	0.3285	0.7196	0.6717	0.6886
Proposed	**0.8173**	**0.0964**	**0.2408**	**0.8186**	**0.7588**	**0.7784**

## Data Availability

The data are not publicly available due to privacy restrictions.
